# Supplementary material for: Nemacol is a small molecule inhibitor of C. elegans vesicular acetylcholine transporter with anthelmintic potential
Source: Nat Commun. 2023 Mar 31;14:1816. doi: 10.1038/s41467-023-37452-6 (PMC10066365; doi:10.1038/s41467-023-37452-6)
Supplement: Supplementary file 1 — Supplementary Information. [file 41467_2023_37452_MOESM1_ESM.pdf]

Supplementary Information for:

Nemacol is a Small Molecule Inhibitor of *C. elegans* Vesicular Acetylcholine Transporter with Anthelmintic Potential

Sean Harrington[1,2,3*], Jacob Pyche[1,2,*], Andrew R. Burns[2,3], Tina Spalholz[4], Kaetlyn T. Ryan[5], Rachel J. Baker[6], Justin Ching[6], Lucien Rufener[7], Mark Lautens[6], Daniel Kulke[8†,‡], Alexandre Vernudachi[9], Mostafa Zamanian[5], Winnie Deuther-Conrad[4], Peter Brust[4,$], and Peter J. Roy[1,2,3,10]

1. Department of Pharmacology and Toxicology, University of Toronto, Toronto, ON, M5S 1A8, Canada
2. The Donnelly Centre for Cellular and Biomolecular Research, University of Toronto, Toronto, ON, M5S 3E1, Canada
3. Department of Molecular Genetics, University of Toronto, Toronto, ON, M5S 1A8, Canada
4. Department of Neuroradiopharmaceuticals, Institute of Radiopharmaceutical Cancer Research, Helmholtz-Zentrum Dresden-Rossendorf, 04318 Leipzig, Germany
5. Department of Pathobiological Sciences, University of Wisconsin-Madison, Madison, WI USA
6. The Department of Chemistry, University of Toronto, 80 St. George Street, M5S 3H6, Toronto, CANADA.
7. INVENesis Sàrl, Route de Neuchâtel 15A, 2072 St Blaise (NE), Switzerland.
8. Research Parasiticides, Bayer Animal Health GmbH, Monheim, Germany.
9. INVENesis France Sàrl, 147 Avenue André Maginot, Tours, 37100 France.

†Current affiliation, Department of Biomedical Sciences; Iowa State University, Ames, Iowa 50011, USA.
‡Current affiliation, Global Innovation, Boehringer Ingelheim Vetmedica GmbH; Binger Str. 173, 55218 Ingelheim am Rhein, Germany.
$Current affiliation, The Lübeck Institute of Experimental Dermatology, University Medical Center Schleswig-Holstein, 23562 Lübeck, Germany

*These authors contributed equally to this work.

[10]Corresponding Author: Peter J. Roy
Tel: (416) 946-8395
Fax: (416) 978-6885
E-mail: peter.roy@utoronto.ca

Note that the citations for the cited literature are noted in the main manuscript.

compound	biological replicate #1 experiment date	Ki (nM; 95% CI)	biological replicate #2 experiment date	Ki (nM; 95% CI)	biological replicate #3 experiment date	Ki (nM; 95% CI)	mean Ki (nM)
(-)vesamicol	30-10-2019	19.5 (17.7 - 21.6)	21-11-2019	22.4 (17.2 - 29.4)			21
nemacol-1	30-10-2019	875 (665 - 5993)	21-11-2019	661 (503 - 868.2)			768
nemacol-5	26-11-2019	380 (326 - 443)	05-12-2019	372 (185 - 747)			376
nemacol-19	26-11-2019	484 (369 - 634)	18-12-2019	439 (295 - 653)			462
nemacol-41	16-06-2020	482 (298 - 780)	30-07-2020	376 (275 - 514)			429
nemacol-43	15-06-2020	191 (169 - 1079)	14-07-2020	126 (102 - 157)			159
nemacol-45	16-06-2020	211 (117 - 380)	25-08-2020	210 (112 - 393)	17-12-2022	236 (167 - 333)	219
nemacol-46	15-06-2020	91.6 (28.4 - 296)	30-07-2020	75.4 (40.6 - 140)			83.5
nemacol-50	14-07-2020	1818 (1124 - 2939)	25-08-2020	2052 (1443 - 2919)	17-12-2022	1945 (1450 - 2609)	1938
nemacol-53	09-07-2020	554 (165 - 1862)	30-07-2020	1042 (655 - 1657)			798
nemacol-59	16-06-2020	1850 (282 - 11950)	22-07-2020	1676 (890 - 3154)			1763
nemacol-62	09-09-2020	2340 (1239 - 4418)	25-09-2020	3342 (2549 - 4381)			2841
nemacol-63	09-09-2020	4893 (2250 - 10640)	25-09-2020	8468 (4790 - 14970)			6681

Supplementary Table 1: Nemacol Analogs rat VAChT K_i Summary Table. Shown K_i values are from binding curves tested over a 10-fold concentration series from 10^{-11} M to 10^{-5} M of each analog over technical triplicate measurement.

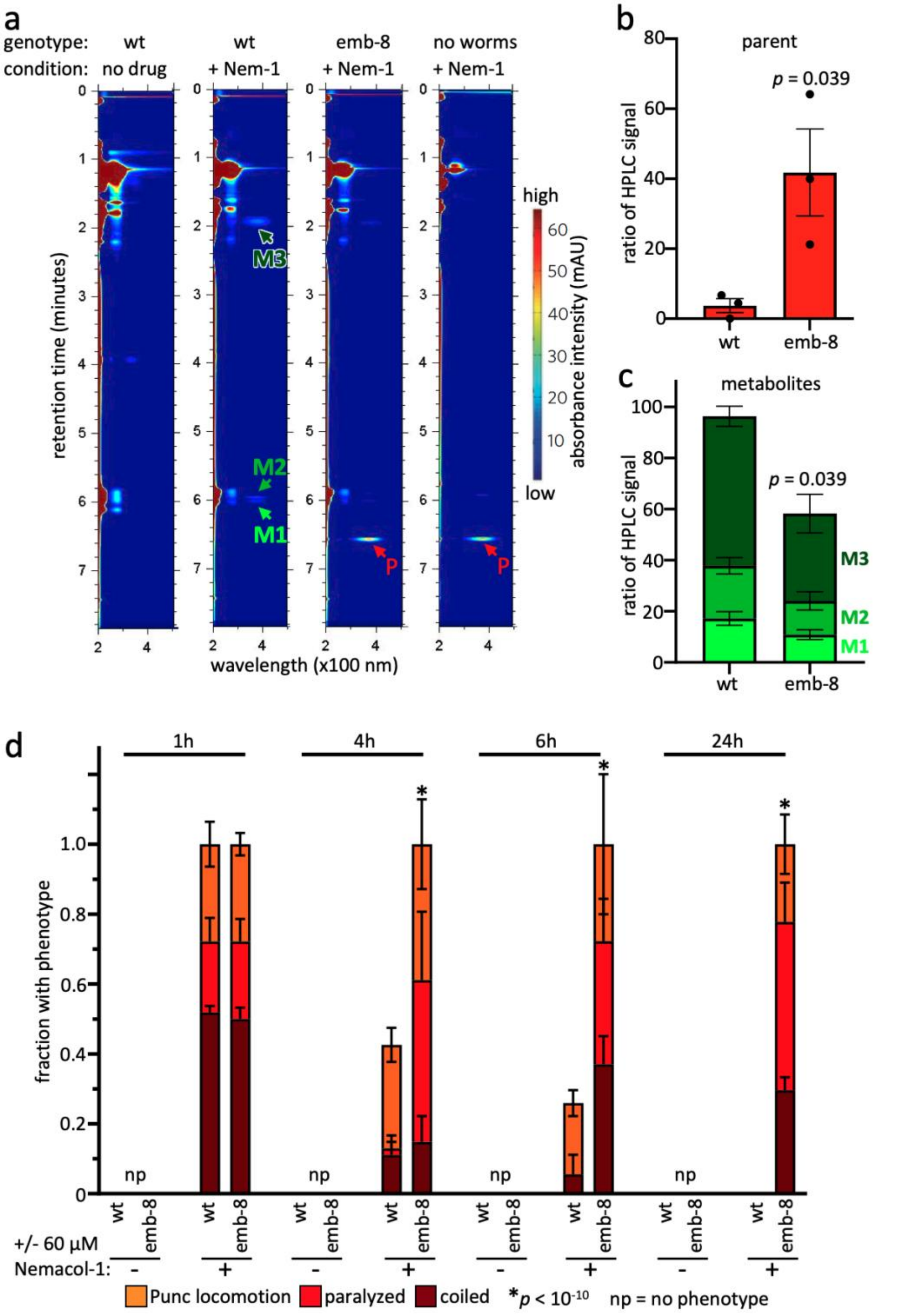

Supplementary Figure 1. Disruption of the EMB-8 Cytochrome p450 Reductase Suppresses *C. elegans*' Ability to Metabolize Nemacol-1 and the Worms' Ability to Recover from Nemacol-1-

induced motor defects. (**a**) Heat-mapped chromatograms of exemplar HPLC runs that are coupled to a diode array detector (HPLC-DAD) of wild-type and EMB-8-knockdown animals treated with solvent-only control or 60 µM Nemacol-1 (Nem-1) after 6 hours of incubation. Retention time is shown on the y axis, and absorbance wavelength is shown on the x axis. The scale of absorbance intensity, in milli–absorbance units (mAU), is shown on the right. The parent (P) Nemacol-1 peak (red arrow), and Nemacol metabolite (M) peaks (green arrows) are indicated. See methods for HPLC-DAD methodology. (**b and c**) Quantification of the relative ratio of parent and metabolite peaks to the total compound related signal from triplicate measurement of 2000 adult worms by HPLC-DAD (see methods). Reporting the mean of three biological replicates (N = 3) with the standard error of the mean. *P* values were generated using unpaired two-sided students t-test. (**d**) EMB-8 disruption suppresses the dissipation of the Nemacol-induced phenotypes. Wild type adult worms were grown on *L4440 control* (RNAi)-inducing bacteria and adult *emb-8(hc69)* mutants were grown on *emb-8*(RNAi)-inducing bacteria, all at 25°C (see methods for more details). Worms were then transferred to solid plates containing 60 µM Nemacol-1 containing the respective RNAi-inducing bacteria and their locomotion was observed over a 24-hour time course. Data are the mean of 3 biological replicates (N = 3) scoring 18 animals (n = 18) per trial reporting the SEM. Statistical comparisons between the emb-8-treatment and wt were made using one-sided Fisher's method combining Fisher Exact Test values comparing the fraction of animals exhibiting any motor phenotype between wt and the *emb-8* samples. A black asterisks indicates *p*<E-10. The *p* values are 1.2E-10 (4 hours), 1.2E-15 (6. Hours), and 7.3E-28 (24 hours).

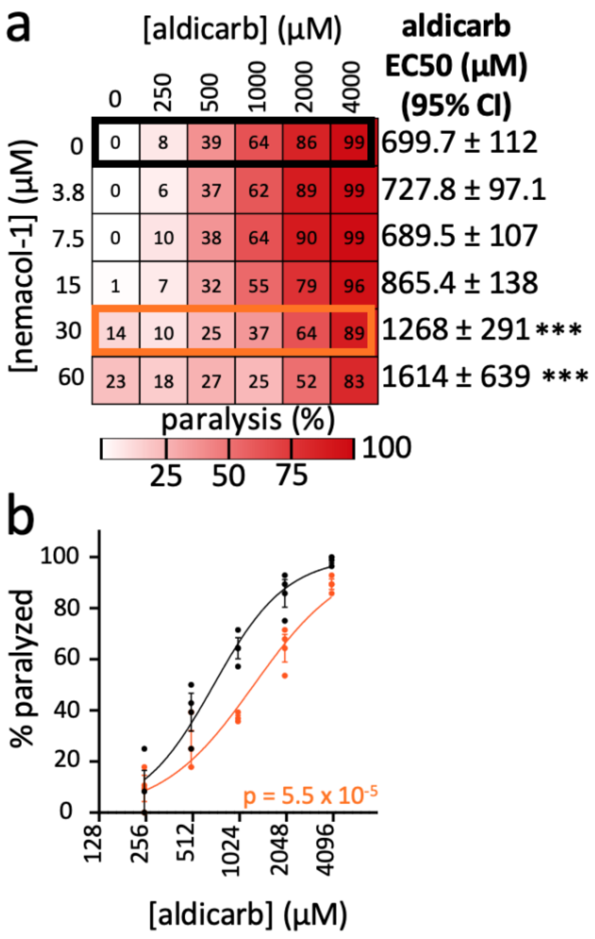

Supplementary Figure 2. Nemacol-1 Suppresses the Paralysis Induced by the Carbamate Acetylcholinesterase Inhibitor Aldicarb. (**a**) Double dose-response matrices of Nemacol-1 + aldicarb showing the fraction of animals that were scored as paralyzed after 80 minutes. The benchmark for paralysis was a lack of sinusoidal body posture and an inability to back at least half a body length upon a touch on the head with a platinum wire. Values in cells represent the percent of animals scored as paralyzed. Data are the mean of 3 biological replicates (N = 3) scoring 28 animals per condition reporting the SEM. P-value calculated using two-sided extra sum-of-squares F test comparing the aldicarb dose response curves with and without Nemacol-1) in GraphPad Prism version 9.3.1. The black asterisks indicate p<0.001. The p-values are 1.0E-

03 (30 μM Nemacol-1) and 8.5E-5 (60 μM Nemacol-1). ((**b**) The dose-response curves from (a) of wild type *C. elegans* animals exposed to increasing concentrations of aldicarb comparing animals with no Nemacol treatment (black line, corresponding the values in the black box in (a)) versus treatment with 30 μM Nemacol-1 (orange line, corresponding the values in the orange box in (a)) (p = 5.5E-5). P-value calculated using two-sided extra sum-of-squares F test comparing the aldicarb dose response curves +/- 30 μM Nemacol-1 in GraphPad Prism version 9.3.1. See (a) for the EC50s.

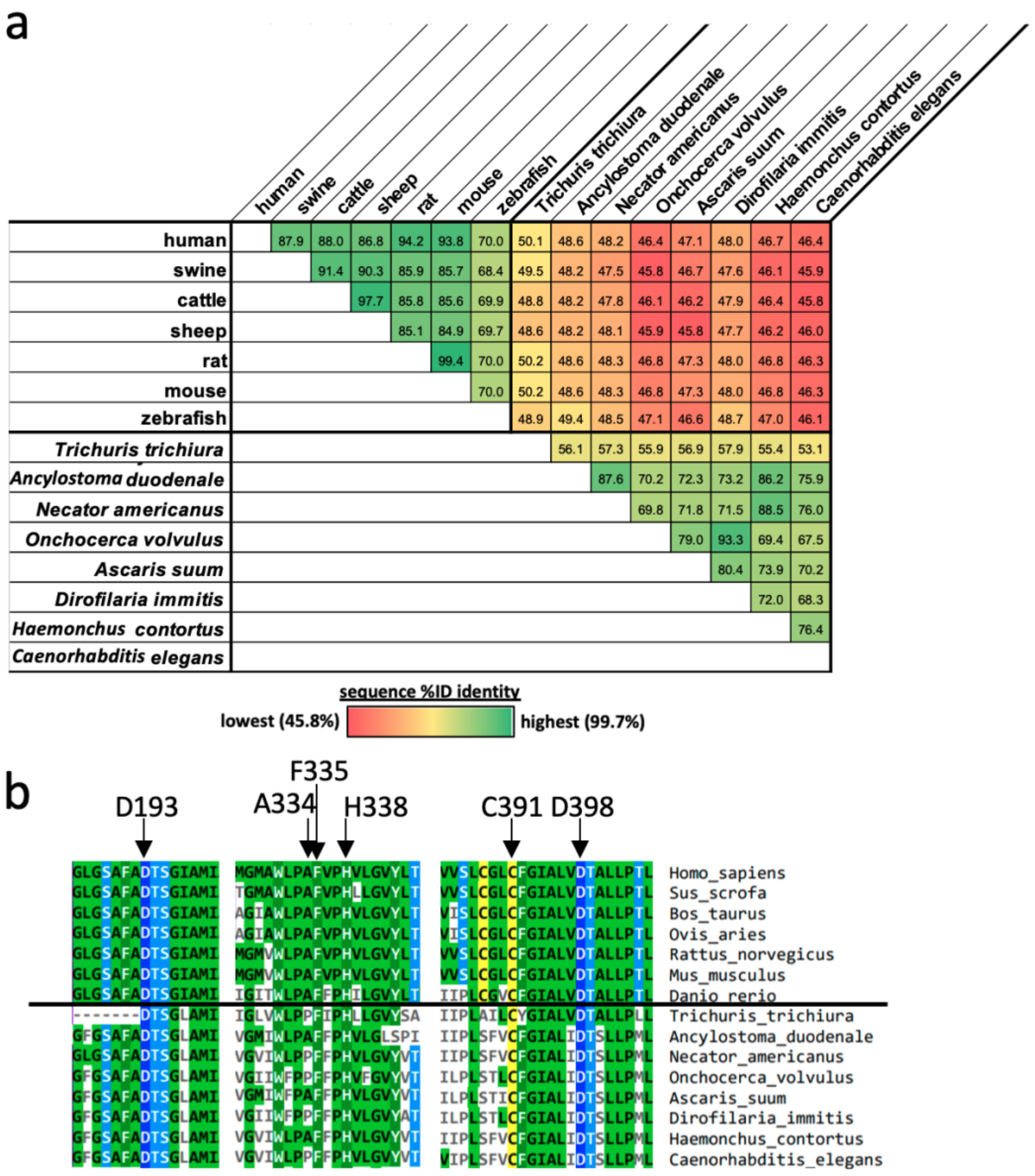

Supplementary Figure 3. VAChT Sequence Differences Exist Between Nematode and Vertebrates. (**a**) VAChT sequence identity matrix comparing the percentage of residues that are shared between each species VAChT sequence. Sequences were identified as the closest homolog to human VAChT identified through BLAST searches[79]. Sequences used (NCBI reference sequence identifiers or otherwise stated): human NP_003046.2 (https://www.ncbi.nlm.nih.gov/protein/118582257); swine (*Sus scrofa*): XP_013838900.2 (https://www.ncbi.nlm.nih.gov/protein/XP_013838900.2); cattle (*Bos taurus*): XP_002699016.1

(https://www.ncbi.nlm.nih.gov/protein/XP_002699016.1); sheep (*Ovis aries*): XP_027818269 (https://www.ncbi.nlm.nih.gov/protein/XP_027818269); mouse (*Mus Musculus):* NP_068358.2 (https://www.ncbi.nlm.nih.gov/protein/NP_068358.2); rat (*Rattus norvegicus):* NP_113851.1 (https://www.ncbi.nlm.nih.gov/protein/NP_113851.1); zebrafish (*Danio rerio*): NP_001071018.1 (https://www.ncbi.nlm.nih.gov/protein/NP_001071018.1); *Trichuris trichiura:* CDW52212.1 (https://www.ncbi.nlm.nih.gov/protein/CDW52212.1); *Ancylostoma duodenale:* KIH66835.1 (https://www.ncbi.nlm.nih.gov/protein/KIH66835.1); *Necator americanus:* XP_013297134.1 (https://www.ncbi.nlm.nih.gov/protein/XP_013297134.1); *Onchocerca volvulus:* A0A2K6VZC1 (https://www.uniprot.org/uniprotkb/A0A2K6VZC1/entry); *Ascaris suum:* AgB02_g088_t01 (https://tinyurl.com/3zhd3wfu); *Dirofilaria immitis:* nDi.2.2.2.t09212 (https://tinyurl.com/29cmv8h8); *Haemonchus contortus:* A0A7I4YIM0 (https://www.uniprot.org/uniprotkb/A0A7I4YIM0/entry); *C. elegans:* NP_001379838.1 (https://wormbase.org/species/c_elegans/protein/CE17307#06--10). (**b**) A multiple sequence alignment of the VAChT sequences shown in panel A that highlights residues that have been reported to decrease Vesamicol affinity when mutated[27,31]. VAChT sequences are separated by vertebrate sequences (top) and nematode sequences (bottom). Showing 7 residues flanking Vesamicol specificity determinants. Residue numbers are the position in the human sequence (D193 & D398[80], A334[34], F335[35], H338[36] and C391[31]).

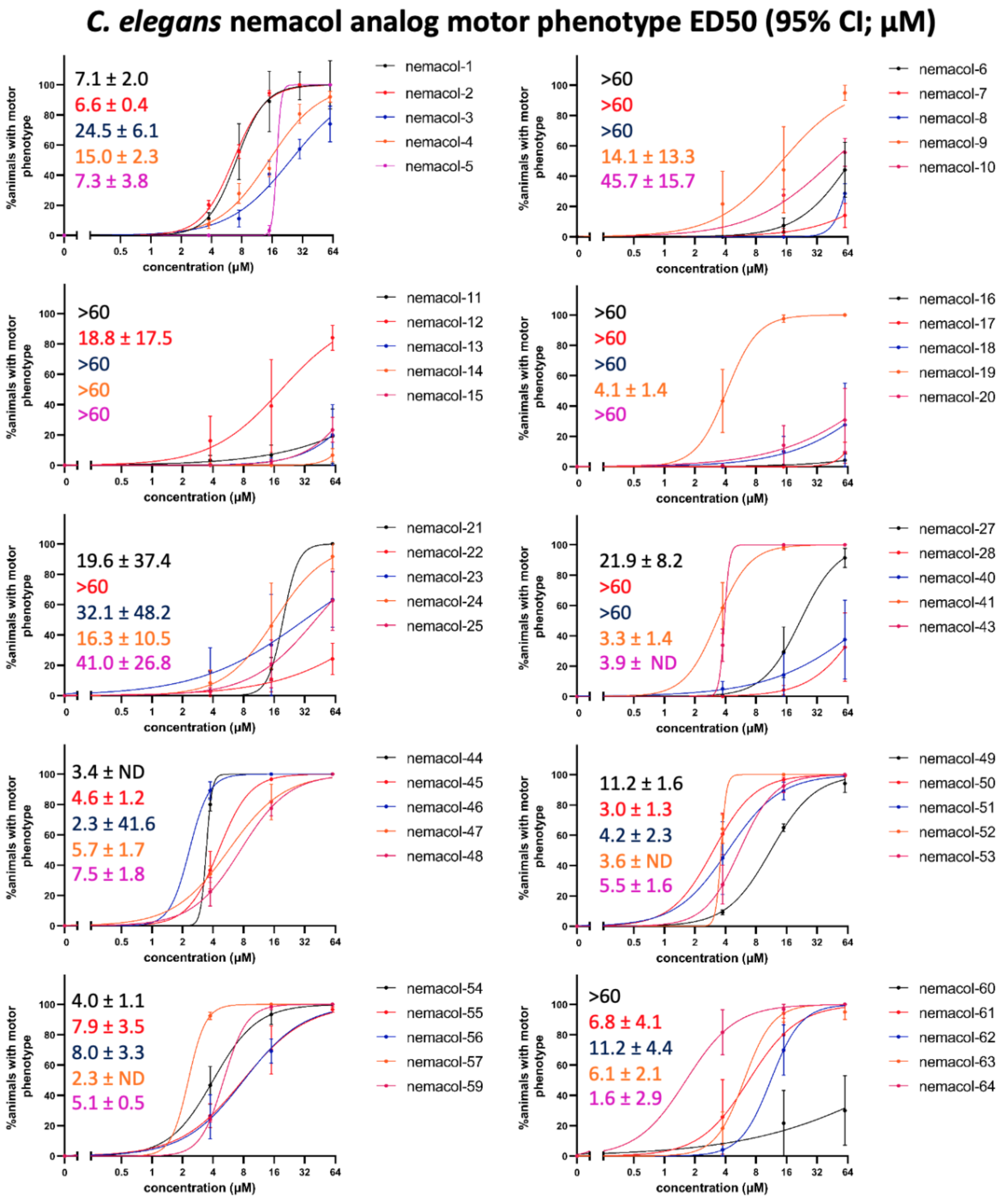

Supplementary Figure 4. Nemacol analogs disrupt *C. elegans* motility in a dose-dependent manner. Showing percentage of *C. elegans* young-adult animals exhibiting any motor defect over a dose-response of each indicated nemacol analog after one hour of exposure on media

seeded with OP50 *E. coli* as a food source (see methods for further details). Data are the mean of three biological replicates (N = 3) scoring 18 animals per trial showing the standard error of the mean. EC50 values and associated symmetrical 95% confidence intervals were calculated from four parameter binding curves were fit using GraphPad Prism (version 9.3.1) and are summarized in Fig. 4c.

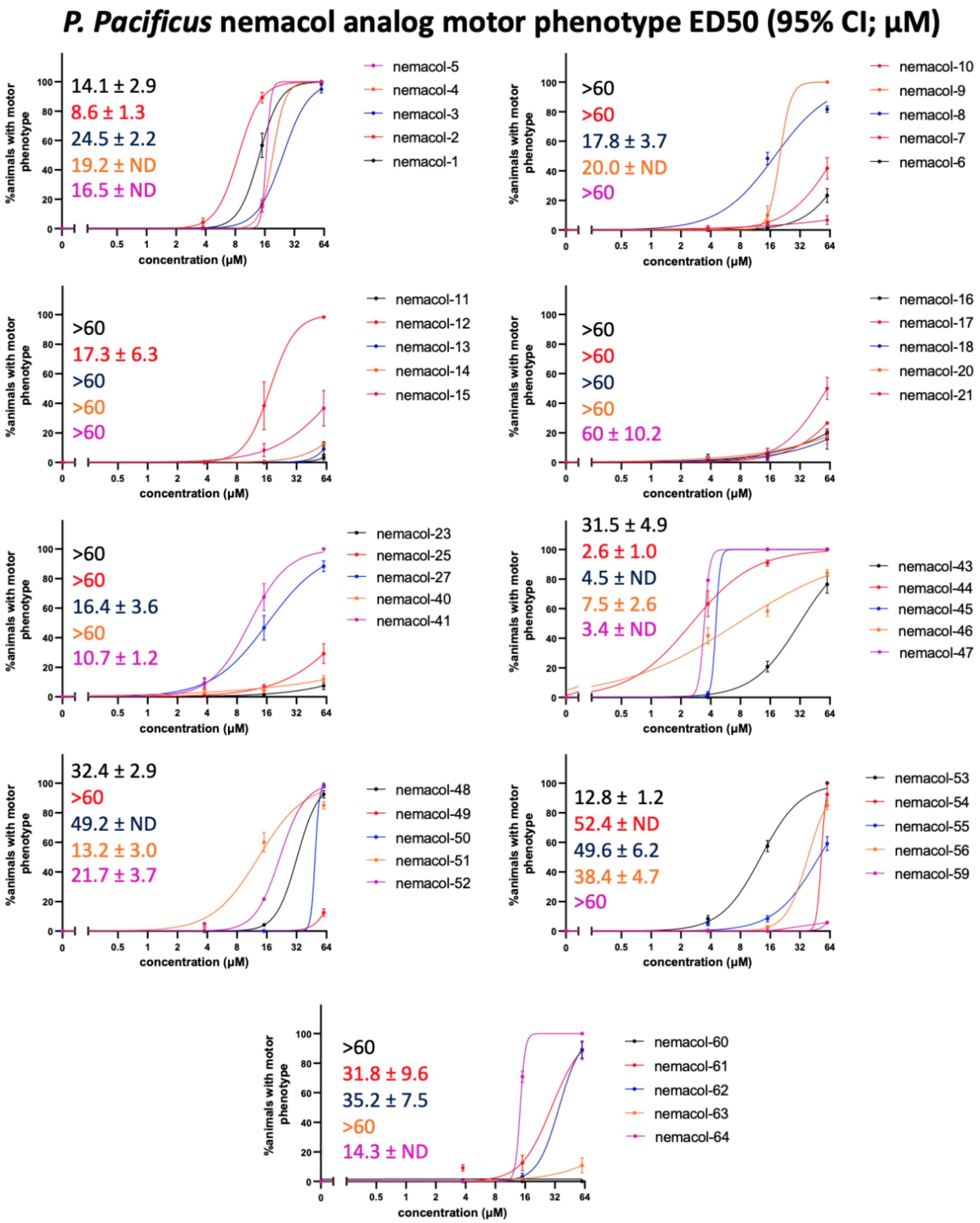

Supplementary Figure 5. Nemacol analogs disrupt *P. pacificus* motility in a dose-dependent manner. Showing the percentage of *P. pacificus* young-adult animals exhibiting any motor

defect over a dose-response of each indicated nemacol analog after one hour of exposure on media seeded with OP50 *E. coli* as a food source (see methods for further details). Data are the mean of three biological replicates (N = 3) scoring 18 animals per trial showing the standard error of the mean. EC50 values and associated symmetrical 95% confidence intervals were calculated from four parameter binding curves were fit using GraphPad Prism (version 9.3.1) and are summarized in Fig. 4c.

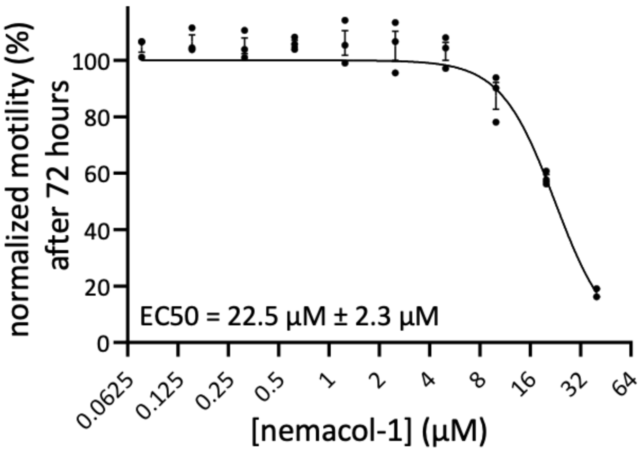

Supplementary Figure 6. Nemacol-1 disrupts *Dirofilaria immitis* motility in a dose-dependent manner. *D. immitis* microfilariae (Missouri isolate) motility over a dose-response of Nemacol-1. Reporting the control-normalized effect on microfilariae motility after 72-hours of incubation in a 96-well plate with compound in 1% DMSO sealed with a breathable plate cover and maintained at 37°C and 5% atmospheric CO_2. The graph shows each replicate from three independent biological replicates (N = 3), testing 500 microfilariae per well, and shows the standard error of the mean (see methods for additional details). The EC50 value and associated symmetrical 95% confidence interval was calculated from four parameter binding curves were fit using GraphPad Prism (version 9.3.1).

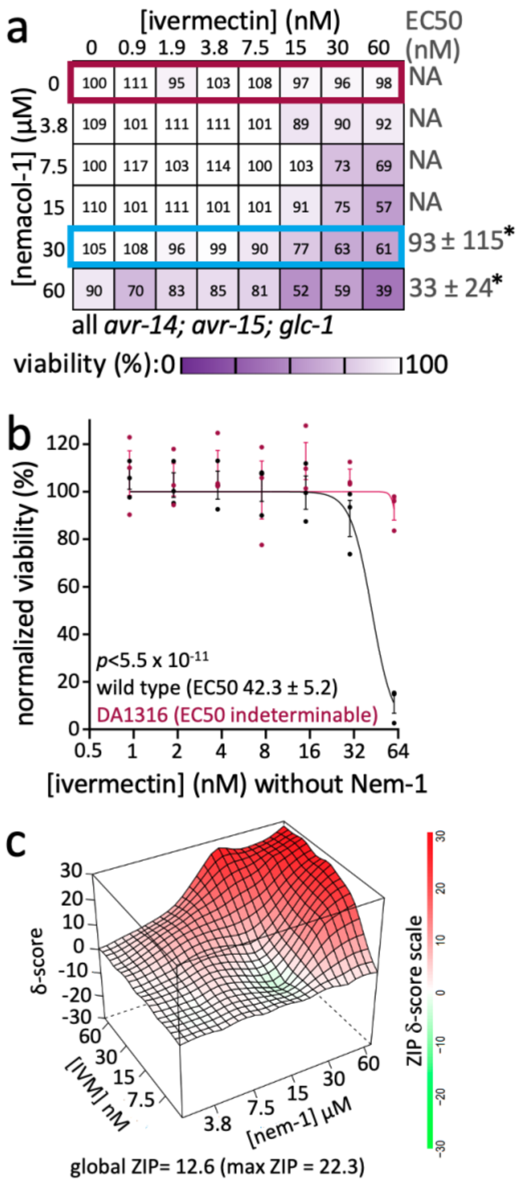

Supplementary Figure 7. Nemacol-1 synergistically kills *C. elegans* in combination with Ivermectin. (a) *C. elegans* DA1316 strain *avr-14(ad1305); avr-15(vu227) glc-1(pk54)* viability over a double dose response matrix of Nemacol-1 and Ivermectin reporting the means of the control-normalized fraction of animals alive in treatment wells of 3-days of growth assays (see methods). Data are from three biological replicates (N = 3) conducted in technical duplicate (n = 2). A black asterisk indicates $p<0.001$ relative to the dose response without Nemacol-1. (**b**)

Dose-response curves highlighting the Ivermectin killing of wild-type *C. elegans* animals without Nemacol-1 treatment (black line, corresponding the values in the black box in Fig. 5a)) versus DA1316 mutants without Nemacol-1 treatment (magenta line, corresponding the values in the magenta box in (a). Data are from three biological replicates (N = 3) conducted in technical duplicate (n = 2) reporting the SEM. The *p*-values for (a) and (b) were calculated using two-sided extra sum-of-squares F test in GraphPad Prism (version 9.3.1). In (a), the *p*-values for comparing the ivermectin dose response in the presence of Nemacol-1 relative to its absence are 2.7E-07 (for 30 μM Nemacol-1) and 5.8E-08 (for 30 μM Nemacol-1). In (b), the *p*-value is 5.5E-11. For (a) and (b), ~20 *C. elegans* L1s were added to wells containing the indicated condition with 0.6% DMSO and an HB101 *E. coli* food source in nematode growth medium. (**c**) Zero Interaction Potency (ZIP) synergy score plot of the Ivermectin + Nemacol-1 double-dose response interaction in DA1316 animals generated using the SynergyFinder2.0 server[42]. The combination yields global ZIP synergy score of 12.6, which is beyond the ZIP score threshold of synergy (10)[33-35].
