# Supplementary material for: Nemacol is a small molecule inhibitor of C. elegans vesicular acetylcholine transporter with anthelmintic potential
Source: Nat Commun. 2023 Mar 31;14:1816. doi: 10.1038/s41467-023-37452-6 (PMC10066365; doi:10.1038/s41467-023-37452-6)
Supplement: Supplementary file 9 — Description of additional supplementary files [file 41467_2023_37452_MOESM9_ESM.pdf]

Description of Additional Supplementary Files for, '*Nemacol is a Small Molecule Inhibitor of C. elegans Vesicular Acetylcholine Transporter with Anthelmintic Potential*'

Title: Supplementary Movie 1

Description: One wild-type young adult locomoting before and after head touch with a platinum wire. The animal was on media containing 1% DMSO (control) with a lawn of OP50 *E. coli* bacteria as a food source for 80 minutes before the movie was shot.

Title: Supplementary Movie 2

Description: Three wild-type young adult animals exhibiting the Punc phenotype and then being prodded on the head after 60 minutes of exposure on media containing 60 µM Nemacol-1 + 1% DMSO with a lawn of OP50 *E. coli* bacteria as a food source.

Title: Supplementary Movie 3

Description: One wild type animal exhibiting the Punc phenotype and being prodded on the head after 80 minutes of exposure on media containing 60 µM nemacol-1 + 1% DMSO with a lawn of OP50 *E. coli* bacteria as a food source.

Title: Supplementary Movie 4

Description: An example of the coiling phenotype. Three wild type animals swimming after 80 minutes of exposure on media containing 60 µM nemacol-1 + 1% DMSO with a lawn of OP50 *E. coli* bacteria as a food source. One animal (at the 11 o'clock position) exhibits the coiling phenotype while the other two exhibit exaggerated body bends.

Title: Supplementary Movie 5

Description: A wild-type animal transiting between Punc-like locomotion to a coiled body posture after 45 minutes of exposure on media containing 60 µM nemacol-1 + 1% DMSO with a lawn of OP50 *E. coli* bacteria as a food source.

Title: Supplementary Movie 6

Description: A wild-type animal exhibiting the 'paralysis' phenotype exhibiting no movement before or after aggressive touch with a platinum wire after 80 minutes of exposure on media containing 60 µM nemacol-1 + 1% DMSO with a lawn of OP50 *E. coli* bacteria as a food source.

Title: Supplementary Movie 7

Description: Another example of a wild-type animal exhibiting the 'paralysis' phenotype exhibiting no movement before or after aggressive touch with a platinum wire after 80 minutes of exposure on media containing 60 µM nemacol-1 + 1% DMSO with a lawn of OP50 *E. coli* bacteria as a food source.
