# Supplementary material for: Nemacol is a small molecule inhibitor of C. elegans vesicular acetylcholine transporter with anthelmintic potential
Source: Nat Commun. 2023 Mar 31;14:1816. doi: 10.1038/s41467-023-37452-6 (PMC10066365; doi:10.1038/s41467-023-37452-6)
Supplement: Supplementary file 10 — Reporting Summary [file 41467_2023_37452_MOESM10_ESM.pdf]

nature portfolio

Corresponding author(s): Peter J. Roy

Last updated by author(s): Mar 8, 2023

Reporting Summary

Nature Portfolio wishes to improve the reproducibility of the work that we publish. This form provides structure for consistency and transparency in reporting. For further information on Nature Portfolio policies, see our Editorial Policies and the Editorial Policy Checklist.

Statistics

For all statistical analyses, confirm that the following items are present in the figure legend, table legend, main text, or Methods section.

n/a	Confirmed	
☐	☒	The exact sample size (*n*) for each experimental group/condition, given as a discrete number and unit of measurement
☐	☒	A statement on whether measurements were taken from distinct samples or whether the same sample was measured repeatedly
☐	☒	The statistical test(s) used AND whether they are one- or two-sided<br>*Only common tests should be described solely by name; describe more complex techniques in the Methods section.*
☒	☐	A description of all covariates tested
☒	☐	A description of any assumptions or corrections, such as tests of normality and adjustment for multiple comparisons
☐	☒	A full description of the statistical parameters including central tendency (e.g. means) or other basic estimates (e.g. regression coefficient) AND variation (e.g. standard deviation) or associated estimates of uncertainty (e.g. confidence intervals)
☐	☒	For null hypothesis testing, the test statistic (e.g. *F*, *t*, *r*) with confidence intervals, effect sizes, degrees of freedom and *P* value noted<br>*Give P values as exact values whenever suitable.*
☒	☐	For Bayesian analysis, information on the choice of priors and Markov chain Monte Carlo settings
☒	☐	For hierarchical and complex designs, identification of the appropriate level for tests and full reporting of outcomes
☒	☐	Estimates of effect sizes (e.g. Cohen's *d*, Pearson's *r*), indicating how they were calculated

Our web collection on statistics for biologists contains articles on many of the points above.

Software and code

Policy information about availability of computer code

Data collection	Custom original code has not been generated for this work. Other methods of data collection are reported in the methods section.
Data analysis	EC50 curves and statistical analyses were performed using GraphPad Prism (version 9.3.1). Interaction Potency (ZIP) synergy scores and plots of double-dose drug responses were generated using the SynergyFinder2.0 server.

For manuscripts utilizing custom algorithms or software that are central to the research but not yet described in published literature, software must be made available to editors and reviewers. We strongly encourage code deposition in a community repository (e.g. GitHub). See the Nature Portfolio guidelines for submitting code & software for further information.

Data

Policy information about availability of data

All manuscripts must include a data availability statement. This statement should provide the following information, where applicable:

- Accession codes, unique identifiers, or web links for publicly available datasets
- A description of any restrictions on data availability
- For clinical datasets or third party data, please ensure that the statement adheres to our policy

Our Data Availability Statement is as follows: Original data for all analyses presented are included in the Supplementary Data File. Sequences used herein include the following (NCBI reference sequence identifiers or otherwise stated): human NP_003046.2; swine (Sus scrofa): XP_013838900.2; cattle (Bos taurus): XP_002699016.1; sheep (Ovis aries): XP_027818269; mouse (Mus Musculus): NP_068358.2; rat (Rattus norvegicus): NP_113851.1; zebrafish (Danio rerio): NP_001071018.1; Trichuris trichiura: CDW52212.1; Ancylostoma duodenale: KIH66835.1; Necator americanus: XP_013297134.1; Onchocerca volvulus: A0A2K6VZC1

(UniProt ID) Ascaris suum: AgB02_g088_t01 (WormBase ParaSite transcript ID); Dirofilaria immitis: nDi.2.2.2.t09212 (WormBase ParaSite transcript ID); Haemonchus contortus: A0A7I4YIM0 (UniProt ID); C. elegans: NP_001379838.1.
We were asked to explain to the editor why original images (of the worms) are available only upon request. The presented images are simply cropped to be able to fit into the figure- there is only trivial differences between the cropped versions and the original photographs.

Human research participants

Policy information about studies involving human research participants and Sex and Gender in Research.

Reporting on sex and gender	We report the following in the methods section: The sex of C. elegans used in the experiments is hermaphroditic. There are no visible markers of sex differentiation in the Dirofilaria or Haemonchus larvae used herein and there is no evidence of drug effects unevenly distributed across larvae. Given the numbers of Dirofilaria and Haemonchus used in the Kulke, Zamanian and INENesis experiments, both sexes were likely well-represented.
Population characteristics	Not applicable.
Recruitment	Not applicable.
Ethics oversight	We report the following in the methods section: Collaborators working with vertebrate hosts of nematode parasites conducted research complying with relevant ethical regulations. Bayer Animal Health GmbH (Monheim, Germany) operated in accordance with the local Animal Care and Use Committee and governmental authorities (LANUV#200/A176 and #200/A154). The Zamanian group sources their Dirofilarial nematodes from the NIH FR3 (BEI Resources) resource center. Animal research at the FR3 complies with all relevant ethical regulations and operates under the University of Georgia IACUC (AUP#: A2019 04-010-Y1-A0). The INVENesis group performs animal experimentations in the Infectiology of Farm, Model, and Wildlife Animals Facility (PFIE, Centre INRAE Val De Loire, D371753). Experimental protocols were designed in compliance with French law (2010/63/EU, 2010; Rural Code, 2018; Decree No. 2013-118, 2013) concerning the use of laboratory animals. Care and euthanasia of animals were practiced according to the national ethical guidelines and approved by the local ethics committee for animal experimentation (Comité d'Ethique en Expérimentation Animale Val de Loire, CEA VdL N°19): APAFIS#17560. The authors are committed to the principles of the 3Rs: reduction, refinement, and replacement of experimental animals.

Note that full information on the approval of the study protocol must also be provided in the manuscript.

Field-specific reporting

Please select the one below that is the best fit for your research. If you are not sure, read the appropriate sections before making your selection.

☒ Life sciences ☐ Behavioural & social sciences ☐ Ecological, evolutionary & environmental sciences

For a reference copy of the document with all sections, see nature.com/documents/nr-reporting-summary-flat.pdf

Life sciences study design

All studies must disclose on these points even when the disclosure is negative.

Sample size	Nearly throughout, three biological (independent) repeats were performed with multiple technical repeats done within each biological trial. This is standard operating procedure in our lab and in the field and are sufficient for drawing conclusions of significance. Any exceptions to sample sizes are noted in the paper.
Data exclusions	No data was excluded from the trials.
Replication	Statistical analyses were performed as described in the manuscript. Nearly throughout, three biological (independent) repeats were performed with multiple technical repeats done within each biological trial. Upon thawing one of the unc-17 mutant strain a few years after the experiment was first done, as explained in our response to the reviews, we did not see an expected phenotype, so we repeated the experiment and excluded the original data. Our response to the review is pasted here: By contrast, when re-examining e795, we do see obvious Uncoordination with large body bends and a few of the animals were coiled upon prolonged observation. This prompted us to redo the analysis of e795 along side wild type controls. We have updated Figure 2a accordingly. None of the conclusions, however, have changed- e795 remains significantly hypersensitive to Nemacol-1 relative to the wild type control ($p<0.001$)- see Figure 2a.
Randomization	Randomization was not performed. Randomization is simply not standard practice in the field of C. elegans genetics.
Blinding	Blinding was performed with experiments done with Dirofilaria immitis and rat VAChT interaction analyses. Otherwise, blinding is simply not standard practice in the field of C. elegans genetics.

Reporting for specific materials, systems and methods

We require information from authors about some types of materials, experimental systems and methods used in many studies. Here, indicate whether each material, system or method listed is relevant to your study. If you are not sure if a list item applies to your research, read the appropriate section before selecting a response.

Materials & experimental systems

n/a	Involved in the study
☒	☐ Antibodies
☐	☒ Eukaryotic cell lines
☒	☐ Palaeontology and archaeology
☐	☒ Animals and other organisms
☒	☐ Clinical data
☒	☐ Dual use research of concern

Methods

n/a	Involved in the study
☒	☐ ChIP-seq
☒	☐ Flow cytometry
☒	☐ MRI-based neuroimaging

Eukaryotic cell lines

Policy information about cell lines and Sex and Gender in Research

Cell line source(s)	Stably transfected PC12 cells expressing ratVAChT were a gift from Dr. Ali Roghani (Texas Tech University, Lubbock, TX, USA).
Authentication	The cell line was authenticated in that the interaction of the positive control (vesamicol) with the cell line behaved as expected.
Mycoplasma contamination	I have no evidence that the cells were tested for mycoplasma contamination.
Commonly misidentified lines (See ICLAC register)	Not applicable.

Animals and other research organisms

Policy information about studies involving animals; ARRIVE guidelines recommended for reporting animal research, and Sex and Gender in Research

Laboratory animals	The following nematodes were used in this study: C. elegans wild type and mutants, the Missouri isolate of Dirofilaria immitis , and Haemonchus contortus (the susceptible isolate, 'Weybridge', UK).
Wild animals	Not applicable.
Reporting on sex	The sex of C. elegans used in the experiments is hermaphroditic. There are no visible markers of sex differentiation in the Dirofilaria or Haemonchus larvae used herein and there is no evidence of drug effects unevenly distributed across larvae. Given the numbers of Dirofilaria and Haemonchus used in the Kulke, Zamanian and INENesis experiments, both sexes were likely well-represented.
Field-collected samples	Not applicable.
Ethics oversight	Collaborators working with vertebrate hosts of nematode parasites conducted research complying with relevant ethical regulations. Bayer Animal Health GmbH (Monheim, Germany) operated in accordance with the local Animal Care and Use Committee and governmental authorities (LANUV#200/A176 and #200/A154). The Zamanian group sources their Dirofilarial nematodes from the NIH FR3 (BEI Resources) resource center. Animal research at the FR3 complies with all relevant ethical regulations and operates under the University of Georgia IACUC (AUP#: A2019 04-010-Y1-A0). The INVENesis group performs animal experimentations in the Infectiology of Farm, Model, and Wildlife Animals Facility (PFIE, Centre INRAE Val De Loire, D371753). Experimental protocols were designed in compliance with French law (2010/63/EU, 2010; Rural Code, 2018; Decree No. 2013-118, 2013) concerning the use of laboratory animals. Care and euthanasia of animals were practiced according to the national ethical guidelines and approved by the local ethics committee for animal experimentation (Comité d'Ethique en Expérimentation Animale Val de Loire, CEA VdL N°19): APAFIS#17560. The authors are committed to the principles of the 3Rs: reduction, refinement, and replacement of experimental animals.

Note that full information on the approval of the study protocol must also be provided in the manuscript.
